# The Tilapia Cyst Tissue Enclosing the Proliferating *Myxobolus bejeranoi* Parasite Exhibits Cornified Structure and Immune Barrier Function

**DOI:** 10.3390/ijms25115683

**Published:** 2024-05-23

**Authors:** Keren Maor-Landaw, Margarita Smirnov, Tamar Lotan

**Affiliations:** 1 Marine Biology Department, The Leon H. Charney School of Marine Sciences, University of Haifa, Mt. Carmel, Haifa 3103301, Israel; keren.maor@live.com; 2 Central Fish Health Laboratory, Department of Fisheries and Aquaculture, Ministry of Agriculture and Rural Development, Nir David 1080300, Israel; ritas@moag.gov.il

**Keywords:** myxozoa, parasite, tilapia, proteomics, keratin, cyst, gills, infection

## Abstract

Myxozoa, a unique group of obligate endoparasites within the phylum Cnidaria, can cause emerging diseases in wild and cultured fish populations. Recently, the myxozoan *Myxobolus bejeranoi* has been identified as a prevalent pathogen infecting the gills of cultured hybrid tilapia, leading to systemic immune suppression and considerable mortality. Here, we employed a proteomic approach to examine the impact of *M. bejeranoi* infection on fish gills, focusing on the structure of the granulomata, or cyst, formed around the proliferating parasite to prevent its spread to surrounding tissue. Enrichment analysis showed increased immune response and oxidative stress in infected gill tissue, most markedly in the cyst’s wall. The intense immune reaction included a consortium of endopeptidase inhibitors, potentially combating the myxozoan arsenal of secreted proteases. Analysis of the cyst’s proteome and histology staining indicated that keratin intermediate filaments contribute to its structural rigidity. Moreover, we uncovered skin-specific proteins, including a grainyhead-like transcription factor and a teleost-specific S100 calcium-binding protein that may play a role in epithelial morphogenesis and cysts formation. These findings deepen our understanding of the proteomic elements that grant the cyst its distinctive nature at the critical interface between the fish host and myxozoan parasite.

## 1. Introduction

Tilapia, belonging to the Cichlidae family, ranks as the second most widely cultivated fish globally [1] and comprises 60% of the total freshwater fish production in Israel [2]. Intensive aquaculture systems of earthen ponds housing all-male hybrids of *Oreochromis niloticus* (Nile tilapia) females and *O. aureus* (Jordan/blue tilapia) males have become widespread worldwide [1,3,4], including in Israel [3,5]. Hybrid tilapia have gained popularity due to their rapid growth, adaptability to various environmental conditions, stress resistance, and efficient reproduction in captivity [1]. However, in Israeli fish ponds, hybrid tilapia are severely affected by the myxozoan parasite *M. bejeranoi*, which infects these fish at a prevalence exceeding 80% [6].

Myxozoa is a vast group of microscopic obligate endoparasites within the phylum Cnidaria [7]. Myxozoans impact both wild and farmed fish populations, causing diseases such as whirling disease and proliferative kidney disease [8]. The complex myxozoan life cycle involves two hosts; a vertebrate, mostly fish, and an invertebrate, mostly a worm [9,10]. Transmission between hosts is achieved by two distinct types of waterborne spores termed actinospores and myxospores [11,12].

Typically, the first contact of myxozoans with fish hosts is through mucosal surfaces, where they might be challenged by various cell types [13,14]. Teleost fish are armed with both innate and adaptive immunity [15,16,17], and they share a large repertoire of immune system cells and molecules with mammals [18,19]. After passing mucosal and epithelial barriers, the myxozoan parasite travels, typically through the bloodstream, to its specific target tissue where it proliferates [20,21,22]. Once the parasite is at the target tissue, the host activates immune mechanisms including immunoactivating and immunosuppressive cytokines [18,23,24]. Another defensive strategy is the formation of a granuloma, or cyst, around the myxozoan plasmodia. The cyst wall encapsulates the parasite by connective and epithelioid tissue layers originating from the host [25], thereby isolating it and preventing its dispersal to surrounding tissues [18,26].

*M. bejeranoi* sporulation site is the gill filament base [27], where it spills its infective sporoplasm. Myxospores are formed in fish within plasmodia, where multicellular proliferative stages occur. The plasmodia undergo intense cell differentiation to generate sporogonic cell stages, culminating in the production of mature spores [28]. *M. bejeranoi* is a highly efficient parasite that is capable of rapid proliferation within its host. Upon infection, *M. bejeranoi* cells express genes, such as histones, that facilitate rapid cell divisions and proper supply of the required energy [29,30]. Further, similar to other parasites [29,31,32], it employs tactics, such as an increase in calreticulin and an arsenal of proteases, to invade host tissues and eventually shut down its immune system [6].

In a recent transcriptomic study, we characterized the temporal progression of *M. bejeranoi* infection and the immune response of hybrid tilapia. While in the gills, the immune system was triggered at a severe infection stage, in the immuno-organs, the head, kidney, and spleen, an immune suppression was observed [6]. Our recent investigations of the interactions between tilapia fish and *M. bejeranoi* have shown that the infection can occur at very early life stages [33] and that the systemic immune suppression induced by the parasite renders the host vulnerable to other opportunistic pathogens [6]. This might be detrimental to tilapia health and, thereby, have a high economic impact on commercial fish farms. Therefore, we investigated the effects of *M. bejeranoi* infection on fish gills using a proteomic approach, reflecting the tissue’s functional status and its dynamic responses to infection, with a particular focus on the cyst walls. Our findings demonstrate a significant impact on the structure of the gills, along with increased oxidative stress and a local up-regulation of the immune response. Additionally, within the cysts, a robust immune reaction in the form of endopeptidase inhibitor activity was evident. Furthermore, we revealed that the structure of the cysts is composed of specific proteins that support a network of keratin intermediate filaments, which provide the cysts with their rigid structure. These findings shed light on fish defense mechanisms as well as on the critical physical interface between the host and its parasite.

## 2. Results

**Figure 1:**
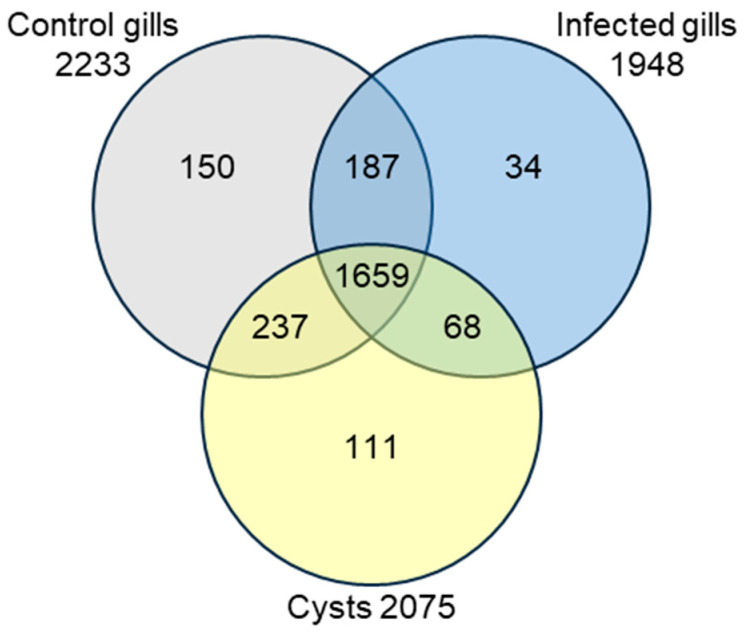
Venn diagram of identified protein groups in healthy control gills, infected gills, and cysts’ proteomes.

To gain insight into the effect of *M. bejeranoi* infection on the gills of hybrid tilapia, we conducted proteomic profiling of three sample types: gill tissue from infected and control uninfected fish and cysts dissected from infected fish. Our analysis identified 2446 protein groups, 1659 of which were shared between the three sample types (Figure 1, Supplementary Table S1).

To identify proteomic changes in gill tissue following infection with *M. bejeranoi*, four biological replicates from control and infected gills were analyzed. Results showed 324 proteins that were significantly differentially expressed between infected and healthy gills, of which 212 were up-regulated and 112 were down-regulated (Welch’s *t*-test, *p* < 0.05) (Supplementary Table S1). To the list of up-regulated proteins, we added 102 proteins that were expressed exclusively in infected tissue, whereas to the list of down-regulated proteins, we added 387 proteins that were found only in control samples (Figure 1).

**Figure 2:**
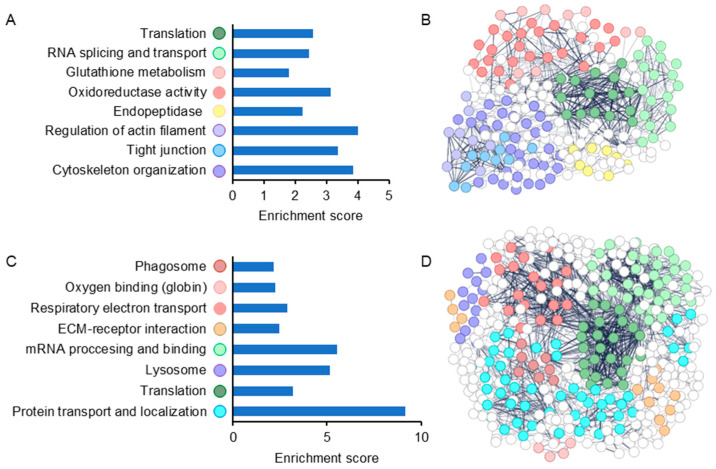
Enrichment analysis of parasite-infected fish gills versus control samples. (**A**,**B**) Up-regulated processes are presented in an enrichment score graph and protein interaction networks (STRING database). (**C**,**D**) Down-regulated processes. Node colors correspond with label in the graph. Details can be found in Supplementary Table S2.

**Figure 3:**
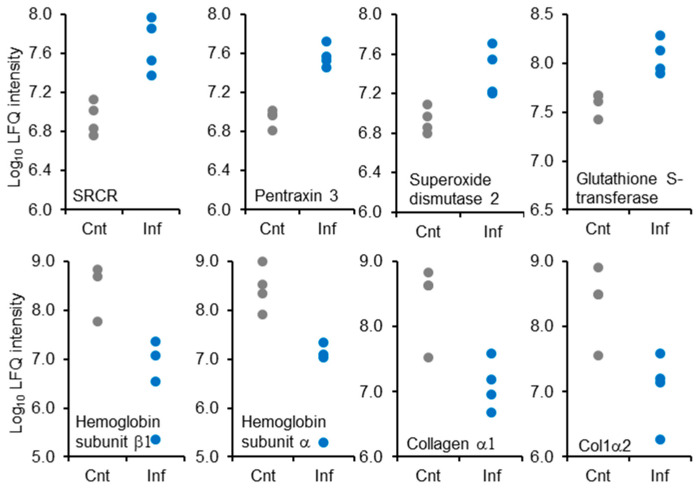
Log_10_ LFQ intensity values of the highest (**top row**) (SRCR, pentraxin 3, superoxide dismutase 2, and glutathione S-transferase-) and the lowest (**bottom row**) (hemoglobin subunit beta1, hemoglobin subunit alpha, collagen alpha-1, and col1α2), ranking proteins in infected gills (Inf) versus control (Cnt) analysis.

Enrichment analysis of up-regulated proteins in infected vs. control gills showed changes in proteins associated with oxidative stress and glutathione metabolism (Figure 2, Supplementary Table S2), among which superoxide dismutase 2 and glutathione S-transferase scored high fold change values (3.42 and 3.13, respectively) (Figure 3, Supplementary Table S1). Other up-regulated clusters contained actin and cytoskeleton and closely related proteins associated with the GO term of tight junction, RNA splicing and transport, and translation (Figure 2, Supplementary Table S2). Following infection with *M. bejeranoi*, proteins related to the fish immune system were increased, most prominently Scavenger Receptor Cysteine-Rich (SRCR) domain-containing protein (m130/cd163) (fold change 6.12) and pentraxin 3 (fold change 4.38) (Figure 3, Supplementary Table S1). The immune response included a notable group of endopeptidase inhibitors, some belonging to the serpin superfamily (Figure 2, Supplementary Table S2).

Enrichment analysis of down-regulated proteins revealed that those related to cellular respiration formed a distinct cluster apart from proteins responsible for oxygen binding (globins) (Figure 2, Supplementary Table S2). Two of the latter category, hemoglobin subunit beta-1 and hemoglobin subunit alpha, had remarkably low fold change values of −68.05 and −35.25, respectively (Figure 3). The next two most extremely down-regulated proteins were two collagen types, alpha-1(I) chain-like (collagen α1) and type I alpha 2 chain (Col1α2), with fold change values of −22.69 and −20.69, respectively. Other down-regulated processes were lysosome, phagosome, and protein transport with a high enrichment score of over 9 (Figure 2).

**Figure 4:**
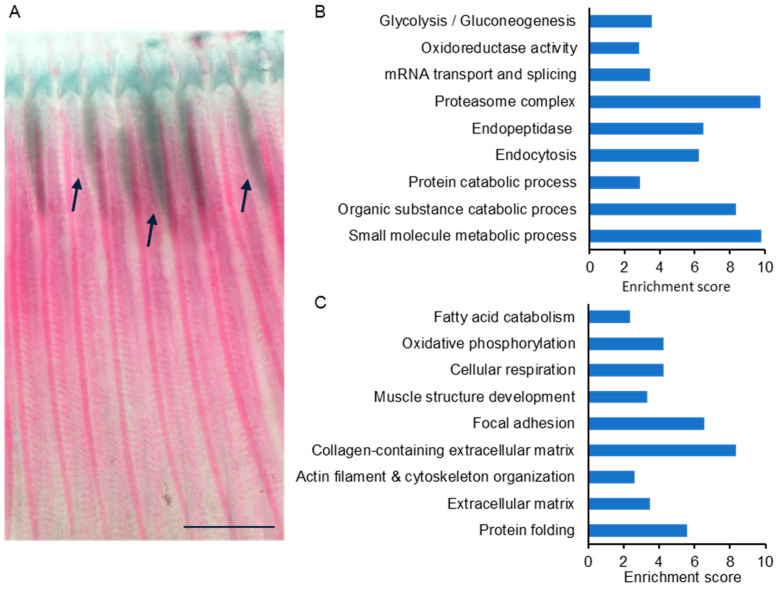
The proteomic profile of cysts. (**A**) Developed cysts (mark with arrows) are localized at the base of the gill filament. Bar 250 μc. (**B**,**C**) Enrichment analysis of cysts versus control samples. Enrichment scores, calculated from adjusted *p*-values, are presented for up-regulated (**B**) and down-regulated processes (**C**). Details can be found in Supplementary Table S3.

To gain insights into the cellular processes occurring at the fish–myxozoan interface, we analyzed the proteomic profile of cysts from infected fish. We sought to identify factors that contribute to the structural integrity of the rigid cysts, isolating their contents from the surrounding fish tissues (Figure 4A). To achieve this goal, we performed proteomic profiling on manually dissected cysts and compared them with the proteomes of gills from healthy, unexposed fish. Two of the analyzed four biological replicates of cyst samples were partial, and therefore, only the other two were used in the analysis. Among the identified 2075 fish protein groups, 179 groups were exclusively expressed in cysts, whereas 337 groups were exclusively expressed in healthy gills (Figure 1). Since gills and cysts are distinct tissues, we employed iBAQ values for the proteomic comparison. These values represent the relative abundance of proteins in each sample [34]. We found 367 protein groups that were significantly up-regulated and 584 groups that were significantly down-regulated in the cyst compared to control gills (Supplementary Table S1). A similar comparison between cysts and control gills was performed using STRING enrichment analysis. Several GO terms that were found for infected gills were also observed in the cyst analysis (Figure 4B, Supplementary Table S3), including oxidoreductase activity and a larger group of endopeptidase inhibitors. Table 1 details the up-regulated proteins within the prominent group of endopeptidase inhibitors, as well as their expression in cysts versus control gills. Similar to infected gills, cysts were also enriched in oxidative-stress-related proteins, with prominent representatives such as glutathione S-transferase and thioredoxin, which had among the highest fold change values (12.12 and 11.13, respectively) (Supplementary Table S1). A distinct cluster of endocytosis and glycolysis, along with proteasome, and catabolic processes, with nearly a 10-fold enrichment, was evident in up-regulated cysts compared to the control. Other highly expressed cyst proteins were the calcium-binding EF-hand domain-containing proteins, ictacalcin and calmodulin 2, with fold change values of 19.63 and 14.27, respectively (Supplementary Table S1). Notably, keratin 98, which is an intermediate filament protein, had a high fold change value of 9.36 in cysts. The pattern of the enriched down-regulated categories in cysts resembled the one observed in infected gills, with oxidative phosphorylation, extracellular matrix, and protein folding. The leading enriched categories were collagen-containing extracellular matrix and focal adhesion (Figure 4C, Supplementary Table S3).

**Figure 5:**
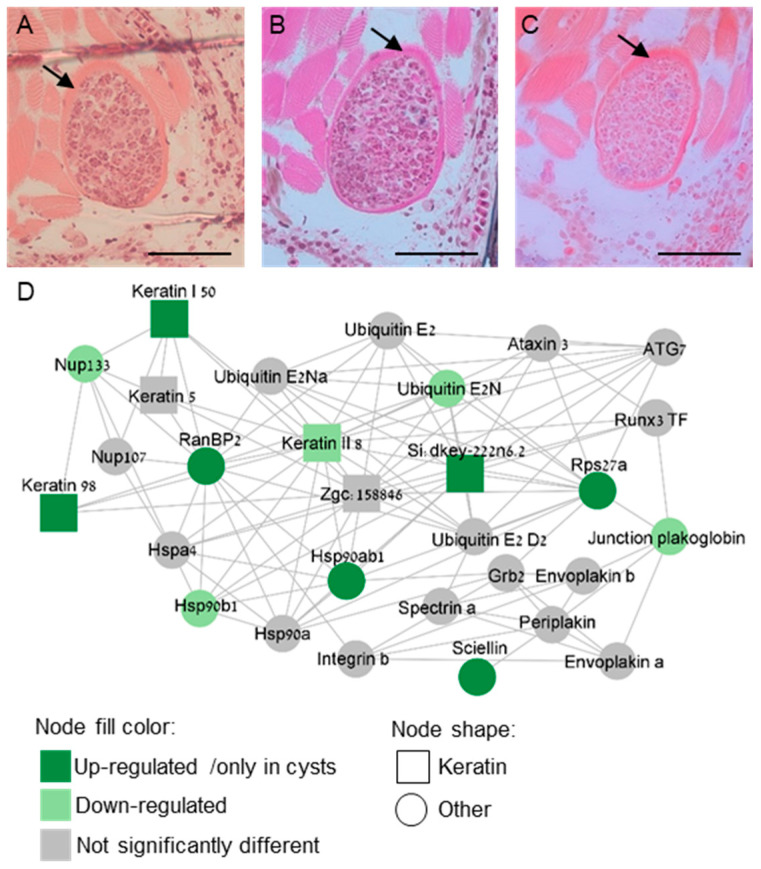
Keratin structure of the cyst’s wall and its protein network. Sections of cysts stained with (**A**) Haematoxylin and eosin, (**B**) Dane-Herman, (**C**) Ayoub-Shklar, showing a keratin-specific staining (mark with arrow); Bar 50 μc. (**D**) Protein interaction network (STRING database) of keratin and sciellin ‘first-neighbors’ that are expressed in the cysts. Intermediate filament proteins are labelled by a square node shape and node fill color is indicative of protein expression pattern.

Next, we mined our proteomic data to identify candidates for structural proteins that could reinforce the barrier formed by the cyst tissue. We therefore focused on differentially expressed proteins in cysts compared to control gill samples, as well as proteins exclusive to cysts. Two proteins involved with cell–cell adhesion were found in cyst proteome and were missing from control samples: cadherin 2 and claudin I, of adherens junction and tight junction, respectively (Supplementary Table S1). We identified three keratin proteins: keratin 98, si:dkey-222n6.2, and keratin, type I cytoskeletal 50 kDa. To visualize keratin localization within the cysts, we applied three different keratin stains, Hematoxylin and Eosin, Ayoub-Shklar, and Dane-Herman stain, all of which are known to effectively stain keratin [35,36]. Our results demonstrate that the cysts’ walls are indeed composed of keratin (Figure 5A–C). Additionally, alongside the identified keratins, we identified two proteins specifically expressed in cysts: sciellin, a precursor of cornified envelope [37] and grainyhead-like transcription factor 1, which plays a role in epidermal barrier structure [38,39,40]. To gain insight into their functions within cysts, we proceeded to explore the protein interactions of these keratins. For this purpose, we generated a protein interaction network of the top 2000 expressed proteins identified within the cysts. A derivative network of keratins and sciellin ‘first-neighbors’ was further drafted and is shown in Figure 5D. The three keratins that were activated in cysts were connected to other cyst-expressed keratins, heat shock proteins, nucleoporins, and several ubiquitins. Sciellin was related to envoplakin, periplakin, and plakoglobin, which are characteristic of cornified epithelia [41] (Figure 5D). Ran binding protein 2 (RanBP2) appeared to be a central node in the network, exhibiting multiple connections. Taken together, the identified protein network indicated a rigid cornified structure of the cyst’s wall.

## 3. Discussion

A common pathological reaction of fish hosts to myxozoan infection is the formation of a granulomatous complex. This cellular barrier encapsulates and isolates the parasite, thereby preventing its spreading to surrounding tissues [18,26]. During the interaction between hybrid tilapia and *M. bejeranoi*, cysts are formed around developing plasmodia in the striated muscle of the gill filament, where infection takes place [27]. While the origin of the cyst tissue is debated [42], a previous study has demonstrated that the cyst walls surrounding the myxozoan are of fish origin [25].

Confined within the cyst walls, a chronic inflammatory reaction involves a complex and diverse array of leukocytes, macrophages, mast cells, and epithelioid cells [18,43]. Upon infection with *M. bejeranoi*, the entire gill tissue exhibits an immune response; however, the intensity of this is higher within the cysts. The SRCR domain-containing protein CD163, which we identified as the most highly expressed immune response protein, exhibited increased expression in cysts. Little is known about SRCR-domain containing proteins in teleosts [44,45]; nonetheless, the essential role of CD163 in the immune system has been demonstrated in humans [46,47]. CD163 is expressed on the surface of macrophages, where its binding to haptoglobin/hemoglobin complexes stimulates macrophage digestion. This process is essential for eliminating free hemoglobin, preventing oxidative stress and cells injury. Additionally, CD163 binding triggers an anti-inflammatory reaction and was thus suggested as a biomarker for inflammation [46,47,48]. Free hemoglobin, the product of hemolysis, can be induced by various pathogens [49], which was recently reported as a feeding behavior of the myxozoan *Sphaerospora molnari* [50,51]. Upon infection, *M. bejeranoi* deploys an array of activated proteases, including cathepsin D and cathepsin L [29], which may act to degrade hemoglobin, as in other parasites [32,52]. This aligns with our findings, indicating decrease in globin proteins and an increase in endocytosis, the process in which macrophages engulf their targets [47].

Myxozoan parasites exploit their host and migrate through its tissues using a battery of secreted proteolytic enzymes [29,32,52,53,54,55]. Thus, a vital component in the host immune arsenal is protease inhibitors [56]. This host–parasite interaction culminates at the cyst walls, further stressing the importance of the cyst in the host strategy to contain the pathogen. Nevertheless, this cellular barrier does not offer absolute protection against the devastating effects of the parasite. Our results reveal an impact on the entire gill tissue manifested, for instance, in pronounced oxidative stress. Moreover, the effects of a progressing infection are systemic, impacting the hematopoietic organs head, kidney, and spleen, leading to immune suppression and high mortality [6].

As sporogenesis progresses, *M. bejeranoi* cells continue to proliferate inside the cysts and the number of oxygen-consuming cells increases, leading to hypoxic conditions [29]. Nevertheless, we did not observe signs of angiogenesis in the host connective tissue, such as in the case of *Myxobolus pendula*, where the host vascular network was suggested to provide the parasite with nutrients and oxygen [42]. This may be related to the changes we reported in the fish cellular respiration.

One of our main questions was related to the protein elements that contribute to the integrity and rigidity of the cyst. Histopathological studies of granulomata forming in fish following exposure to various pathogens, including myxozoans, describe layers of fibroblasts intermingled with collagen fibers and densely packed epithelioid cells [25,42,43,57,58,59,60]. Our proteomic results did not confirm the histological evidence for the presence of collagen. Rather, we observed a substantial down-regulation of collagen in both infected tissue and cysts. This was accompanied by alterations in extracellular matrix organization, either due to the overall stress response or to enable cyst expansion.

Conversely, the elevated levels of keratins we observed align with myxozoan studies that employed anti-keratin antibodies for immunostaining in epidermal and epithelioid cell layers [25,43]. Keratins are a vast family of intermediate filaments that can aggregate into bundles of up to 12 nm in diameter [61], allowing them to endure mechanical forces from neighboring cells and uphold the integrity of the cytoskeletal [62]. Further, there are indications that complexes such as those consisting of cadherin-2 and claudin, which were up-regulated in cysts, play a crucial role in this structure of mechanical stability [63]. The stringent cysts that form in *M. bejeranoi*-infected fish physically press on the local nerves and muscle tissue of the gill filament base [6].

Sciellin, which was expressed only in cysts, is a protein regulator of keratinocytes differentiation and is found in cornified tissue [64,65]. Evoplakin and periplakin, that are apparent in the network, are recruited during this differentiation as well and are associated with keratin filament scaffold formation [41]. The transcription factor grainyhead like 1, which was specific to the cysts, plays a role in epithelial morphogenesis and wound repair [38,39,40]. In gilthead seabream, grainyhead transcription factor 1 along with keratin, are considered markers for skin regeneration [39]. Additionally, keratins are known to be regulated by post-translational modifications, including sumoylation [66]. Possibly RanBP2, which has a SUMO1 E3-like activity [67] and was highly connected to the keratin network in the cyst, plays a role in mediating the assembly of newly formed keratin bundles [68].

Another highly expressed protein in cyst walls was ictacalcin, also known as S100I [69]. This new member in the S100 calcium-binding protein family is exclusively found in teleosts [69]. Ictacalcin is considered to be skin-specific in fish, sometimes related to keratinocytes [70,71]. Its levels were up-regulated in fish infected with amoeba [72] and sea lice [73] and were down-regulated after vaccination with attenuated bacteria [74]. However, S100 proteins are poorly understood in fish. In mammals, they play roles in calcium signaling and inflammatory processes [69,75] and are shown to associate with dental and skin cysts [76,77,78]. Nevertheless, the function of ictacalcin in cysts of hybrid tilapia following *M. bejeranoi* infection remains unclear.

To conclude, our proteomic results indicate that fish cells construct the cyst walls, reinforced with keratin intermediate filaments, producing a rigid structure. At this cellular barrier between the rapidly proliferating myxozoan and its host, the interplay between the fish defense mechanisms and the parasite’s efforts to evade and overcome them is at its highest. However, although the parasite is confined within the cyst, this barrier is often breached, and the infection affects the entire gill tissue, leading to cellular respiratory and oxidative stress. Eventually, the impact will reach the immune organs, resulting in an immune-suppressed fish.

## 4. Materials and Methods

### 4.1. Gill Isolation

Exposure of naïve hybrid tilapia (*O. niloticus* × *O. aureus*) to *M. bejeranoi*-infected fish pond was conducted on August 2021, as we previously reported [29]. Briefly, healthy hybrid tilapia fish with a mean weight of 2.98 g were exposed to the pond water for 24 h using confined cages of ~100 L. The mean water temperature, which was recorded constantly during the experiment using a temperature data logger (HOBO), was 30.25 °C. The fish were then relocated to indoor tanks at the Central Fish Health Laboratory in Nir David. Tanks had a flow-through system with dechlorinated tap water at a temperature of ~25 °C. Fish were sampled at 10- and 20-days post-exposure. Following euthanasia using 1 mL/L 2-phenoxyethanol, gill tissue was dissected from control and infected fish, snap-frozen in liquid nitrogen, and kept at −80 °C until used.

### 4.2. Cyst Isolation

Intact cysts were dissected from infected gills under a Zeiss Discovery V8 binocular (Zeiss, Jena, Germany), using sterilized syringe needles. About 20 isolated cysts from different fish were pooled together into an Eppendorf tube in four biological replicates, snap-frozen in liquid nitrogen and kept at −80 °C until protein extraction.

### 4.3. DNA and Protein Extractions

Four frozen gill lamellae from control or infected fish were dissected under a Zeiss Discovery V8 binocular. Half of the lamellae were transferred into TRIzol reagent (Thermo Scientific, Waltham, MA, USA) for DNA extraction, while the remaining half were placed into a protein extraction buffer containing 0.5% deoxycholate, 10 mM Tris (pH 7.0), and 2 mM EDTA. DNA was extracted according to TRIzol reagent manufacturer’s instructions and as previously reported [6]. Briefly, the tissue was lysed in TRIzol reagent, and the DNA phase was separated using chloroform. The concentration of DNA was measured using a NanoDrop 2000c spectrophotometer (Thermo Scientific, Waltham, MA, USA).The presence of *M. bejeranoi* infection in the fish gills was determined by qPCR using specific primers targeted to amplify the *M. bejeranoi* small subunit ribosomal RNA gene (SSU rDNA), as previously reported [6]. Protein samples from gills and isolated cysts were homogenized in protein extraction buffer using 3 mm glass beads (CS Chemicals Ltd., Ahmedabad, India) in a TissueLyser II (Qiagen, Hilden, Germany) for 3 min at 30 Hz. Samples were then centrifuged, and the protein supernatants were kept at −80 °C until use. For proteolysis, the samples were reduced with 3 mM DTT (54 °C for 45 min), modified with 9 mM iodoacetamide in 400 mM ammonium bicarbonate (in the dark at room temperature for 30 min) and digested with modified trypsin (Promega) at a 1:50 enzyme-to-substrate ratio overnight at 37 °C. A second trypsinization was performed for 4 h. The resulting peptides were acidified with 1% formic acid and precipitated, whereas the peptides in the supernatant were desalted using C18 Stage Tips, dried and re-suspended in 0.1% formic acid.

### 4.4. Mass Spectrometry Analysis

The peptides were resolved by reverse-phase chromatography on 0.075 × 180 mm fused silica capillaries (J&W) packed with Reprosil reversed phase material (Dr Maisch GmbH, Germany). The peptides were eluted with the following concentrations of acetonitrile with 0.1% of formic acid in water at a flow rate of 0.15 μL/min: a linear 180 min gradient of 5 to 28%, followed by a 15 min gradient of 28 to 95% and 25 min at 95% acetonitrile. Mass spectrometry was performed by Q Executive HFX mass spectrometer (Thermo) in a positive mode (*m*/*z* 350–1200, resolution 120,000 for MS1 and 15,000 for MS2) using repetitively full MS scan. This was followed by collision-induced dissociation (HCD, at 27 normalized collision energy) of the 30 most dominant ions (>1 charges) selected from the first MS scan. The AGC settings were 3 × 106 for the full MS and 1 × 105 for the MS/MS scans. A dynamic exclusion list was enabled with an exclusion duration of 20 s. The mass spectrometry proteomic data have been deposited to the ProteomeXchange Consortium via the PRIDE partner repository [79] with the dataset identifier PXD051329.

### 4.5. Gills and Cyst Analysis

The mass spectrometry data were analyzed using the MaxQuant software 2.1.3.0 [80] for peak picking and identification. We used the Andromeda search engine against the *O. niloticus* databases (Ensembl release 110; https://ftp.ensembl.org/pub/release-110/fasta/oreochromis_niloticus (accessed on 12 September 2023)), with mass tolerance of 6 ppm for the precursor masses and the fragment ions. Oxidation on methionine and protein N-terminus acetylation were accepted as variable modifications, and carbamidomethyl on cysteine was accepted as static modifications. Minimal peptide length was set to seven amino acids, and a maximum of two miscleavages were allowed. The data were quantified by label-free analysis using the same software. False discovery rates (FDRs) for peptide and protein levels were filtered to 1% using the target-decoy strategy. Protein tables were filtered to eliminate the identifications from the reverse database and common contaminants. Protein groups were filtered according to the number of unique peptides in each group, with a minimum of one unique peptide in all replicates. Downstream proteomic analyses were performed using Perseus 2.0.11 [81,82]. LFQ intensity values were utilized for the comparison of infected versus control gill samples using Welch’s *t*-test in Perseus 2.0.11 [81,82]. Percent iBAQ values were calculated for cyst and control gill protein groups and further used for Welch’s *t*-test in Perseus 2.0.11 [81,82]. STRING v12.0 [83] was utilized for enrichment analysis and to produce protein interaction networks of significantly and uniquely expressed protein groups (*p* < 0.05) between infected and control gills and between cysts and control gills. An enrichment score was calculated for each group of GO/KEGG/STRING cluster terms, which was defined as the minus log of the geometric mean of all p-values of the GO categories within the group [84]. A STRING protein interaction network of cysts-expressed proteins was constructed using the top 2000 expressed proteins identified within the cysts. A derivative network of keratins and sciellin ‘first-neighbors’ was further drafted and was graphically edited in Cytoscape [85]. A Venn diagram representing the filtered protein groups was drawn using Venny 2.1 (https://bioinfogp.cnb.csic.es/tools/venny/, accessed on 12 September 2023).

### 4.6. Keratin Staining

Gills dissected from infected gills were fixed in 10% neutral-buffered formalin, dehydrated in a graded ethanol series, and embedded in 2-hydroxyethyl methacrylate. Then, 3 μm thick sections were generated using a Leica RM 2245 microtome (Leica Biosystems, Nussloch, Germany). Sections were stained with haematoxylin and eosin, Ayoub-Shklar, and Dane-Herman methods [35,36].

## Figures and Tables

**Table 1:** Endopeptidase inhibitors in cyst wall. Fold change in significant iBAQ up-regulated proteins are shown for cyst versus control. Proteins that are expressed only in cysts and absent in control samples are indicated as ‘cyst only’.

Protein ID	Protein Name	Fold Change
ENSONIP00000013827.2	fetuin-B	cyst only
ENSONIP00000042223.1	alpha-2-HS-glycoprotein 2	cyst only
ENSONIP00000023717.1	kunitz-type protease inhibitor 1	cyst only
ENSONIP00000074191.1	alpha-macroglobulin receptor-binding domain	cyst only
ENSONIP00000067950.1	alpha-2-macroglobulin-like	cyst only
ENSONIP00000005221.1	alpha-2-antiplasmin	cyst only
ENSONIP00000018223.2	inter-alpha-trypsin inhibitor heavy chain 3	cyst only
ENSONIP00000022637.2	leukocyte elastase inhibitor	7.71
ENSONIP00000025380.1	serpin family A member 10	4.70
ENSONIP00000056451.1	legumain	4.15
ENSONIP00000046993.1	cystatin-B	2.51
ENSONIP00000043069.1	cystatin fetuin-A-type domain	2.15
ENSONIP00000001504.1	alpha-1-antitrypsin homolog	1.45
ENSONIP00000009801.2	serpin family C member 1	1.43

## Data Availability

The data supporting the findings of this study are presented in the main text and its additional files. The mass spectrometry proteomic data have been deposited to the ProteomeXchange Consortium via the PRIDE partner repository with the dataset identifier PXD051329.

## Supplementary Materials

The following supporting information can be downloaded at: https://www.mdpi.com/article/10.3390/ijms25115683/s1.
